# An Extensive Meta-Metagenomic Search Identifies SARS-CoV-2-Homologous Sequences in Pangolin Lung Viromes

**DOI:** 10.1128/mSphere.00160-20

**Published:** 2020-05-06

**Authors:** Lamia Wahba, Nimit Jain, Andrew Z. Fire, Massa J. Shoura, Karen L. Artiles, Matthew J. McCoy, Dae-Eun Jeong

**Affiliations:** a Department of Pathology, Stanford University School of Medicine, Stanford, California, USA; b Department of Genetics, Stanford University School of Medicine, Stanford, California, USA; c Department of Bioengineering, Stanford University, Stanford, California, USA; University of Michigan—Ann Arbor

**Keywords:** COVID, SARS-nCoV-2, bioinformatics, coronavirus, metagenomics, pangolin

## Abstract

In numerous instances, tracking the biological significance of a nucleic acid sequence can be augmented through the identification of environmental niches in which the sequence of interest is present. Many metagenomic data sets are now available, with deep sequencing of samples from diverse biological niches. While any individual metagenomic data set can be readily queried using web-based tools, meta-searches through all such data sets are less accessible. In this brief communication, we demonstrate such a meta-metagenomic approach, examining close matches to the severe acute respiratory syndrome coronavirus 2 (SARS-CoV-2) in all high-throughput sequencing data sets in the NCBI Sequence Read Archive accessible with the “virome” keyword. In addition to the homology to bat coronaviruses observed in descriptions of the SARS-CoV-2 sequence (F. Wu, S. Zhao, B. Yu, Y. M. Chen, et al., Nature 579:265–269, 2020, https://doi.org/10.1038/s41586-020-2008-3; P. Zhou, X. L. Yang, X. G. Wang, B. Hu, et al., Nature 579:270–273, 2020, https://doi.org/10.1038/s41586-020-2012-7), we note a strong homology to numerous sequence reads in metavirome data sets generated from the lungs of deceased pangolins reported by Liu et al. (P. Liu, W. Chen, and J. P. Chen, Viruses 11:979, 2019, https://doi.org/10.3390/v11110979). While analysis of these reads indicates the presence of a similar viral sequence in pangolin lung, the similarity is not sufficient to either confirm or rule out a role for pangolins as an intermediate host in the recent emergence of SARS-CoV-2. In addition to the implications for SARS-CoV-2 emergence, this study illustrates the utility and limitations of meta-metagenomic search tools in effective and rapid characterization of potentially significant nucleic acid sequences.

**IMPORTANCE** Meta-metagenomic searches allow for high-speed, low-cost identification of potentially significant biological niches for sequences of interest.

## Abstract (precis)

Meta-metagenomic searches allow for high-speed, low-cost identification of potentially significant biological niches for sequences of interest.

## OBSERVATION

In the early years of nucleic acid sequencing, aggregation of the majority of published DNA and RNA sequences into public sequence databases greatly aided biological hypothesis generation and discovery. Search tools capable of interrogating the ever-expanding databases were facilitated by creative algorithm development and software engineering and by the ever-increasing capabilities of computer hardware and the Internet. In the early 2000s, sequencing methodologies and computational technologies advanced in tandem, enabling quick homology results from a novel sequence without substantial cost.

With the development of larger-scale sequencing methodologies, the time and resources to search all extant sequence data became untenable for most studies. However, creative approaches involving curated databases and feature searches ensured that many key features of novel sequences remained readily accessible. At the same time, the nascent field of metagenomics began, with numerous studies highlighting the power of survey sequencing of DNA and RNA from samples as diverse as the human gut and Antarctic soil (1, 2). As the diversity and size of such data sets expand, the utility of searching them with a novel sequence increases. Meta-metagenomic searches are currently underutilized. In principle, such searches would involve direct access to sequence data from a large set of metagenomic experiments on a terabyte scale, along with software able to search for similarity to a query sequence. We find that neither of these aspects of meta-metagenomic searches is infeasible with current data transfer and processing speeds. In this communication, we report the results of searching the recently described severe acute respiratory syndrome coronavirus 2 (SARS-CoV-2) sequence through a set of metagenomic data sets with the “virome” tag.

## 

### Experimental procedures.

**(i) Computing hardware.** A Linux workstation used for the bulk analysis of metagenomic data sets employs an 8-core i7 Intel microprocessor, 128 gigabyte (GB) of random access memory, 12 terabytes (TB) of conventional disk storage, and 1 TB of solid state drive (SSD) storage. Additional analyses of individual alignments were conducted with standard consumer-grade computers.

**(ii) Sequence data.** All sequence data for this analysis were downloaded from the National Center for Biotechnology Information (NCBI) website, with individual sequences downloaded through a web interface and metagenomic data sets downloaded from the NCBI Sequence Read Archive (SRA) using the SRA-tools package (version 2.9.1). The latter sequence data were downloaded as .sra files using the prefetch tool, with extraction to readable format (.fasta.gz) using the NCBI fastq-dump tool. Each of these manipulations can fail some fraction of the time. Obtaining the sequences can fail due to network issues, while extraction in readable format occasionally fails for unknown reasons. Thus, we developed a set of scripts implementing a workflow that continually requests .sra files with ncbi-prefetch until at least some type of file is obtained, followed by attempts to unpack into .fasta.gz format until one such file is obtained from each .sra file. Metagenomic data sets for analysis were chosen through a keyword search of the SRA descriptions for “virome” and downloaded between 27 January 2020 and 31 January 2020. We note that the “virome” keyword search will certainly not capture every metagenomic data set with viral sequences, and likewise not capture every virus in the short sequence read archive. Despite these clear limitations, the “virome” keyword search identified a broad and diverse set of experimental data sets for further analysis. With up to 16 threads running simultaneously, total download time (prefetch) was approximately 2 days. Similar time was required for conversion to gzipped fasta files. A total of 9,014 sequence data sets were downloaded and converted to fasta.gz files. Most files (as expected) contained large numbers of reads, while a small fraction contained very little data (only a few reads or reads of at most a few base pairs). The total data set consists of 2.5 TB of compressed sequence data corresponding to approximately 10^13^ bases.

**(iii) Search software.** For rapid identification of close matches among large numbers of metagenomic reads, we used a simple dictionary based on the SARS-CoV-2 sequence (our query sequence for SARS-CoV-2 was GenBank accession no. MN908947.3) and its reverse complement, querying every 8th k-mer along the individual reads for matches to the sequence. As a reference, and to benchmark the workflow further, we included several additional sequences in the query (vaccinia virus, an arbitrary segment of an influenza virus isolate, the full sequence of bacteriophage P4, and a number of putative polinton sequences from Caenorhabditis briggsae). The relatively small group of k-mers being queried (<10^6^) allows a rapid search for homologs. This was implemented in a Python script run using the PyPy accelerated interpreter. We stress that this is by no means the most comprehensive or fastest search for large data sets. However, it is more than sufficient to rapidly find any closely matching sequence (with the downloading and conversion of the data, rather than the search, being rate limiting). While the high degree of conservation between isolates of individual coronaviruses and between related coronaviruses (3, 4) facilitates this very simple approach to pattern matching for discovery of closely related viruses in this family, alternative approaches with more-sensitive matching algorithms for other gene or virus families would require only minimally expanded computing resources (5).

**(iv) Alignment of reads to SARS-CoV-2.** Reads from the pangolin hit data sets were adapter rimmed with cutadapt (version 1.18) (6), and mapped to the SARS-CoV-2 genome with BWA-MEM (version 0.7.12) (7) using default settings for paired-end mode. Alignments were visualized with the Integrated Genomics Viewer (IGV) tool (version 2.4.10) (8).

**FIG 1:**
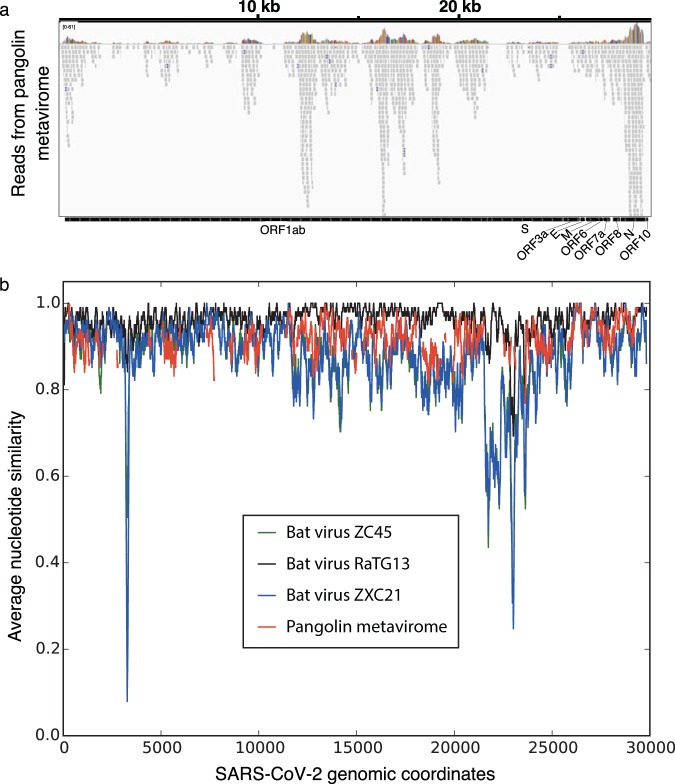
(a) Integrated Genomics Viewer (IGV) snapshot of alignment. Reads from the pangolin lung virome samples (SRA accession no. SRR10168377, SRR10168378, and SRR10168376) were mapped to a SARS-CoV-2 reference sequence (GenBank accession no. MN908947.3). The total numbers of aligned reads from the three samples were 1,107, 313, and 32 reads, respectively. Figure S1 in the supplemental material shows an enlarged view for these alignments within the spike RBD region. (b) Quantification of nucleotide-level similarity between the SARS-CoV-2 genome and pangolin lung metavirome reads aligning to the SARS-CoV-2 genome. Average similarity was calculated in 101-nucleotide windows along the SARS-CoV-2 genome and is only shown for those windows where each nucleotide in the window had coverage of ≥2. Average nucleotide similarity calculated (in 101-nucleotide windows) between the SARS-CoV-2 genome and reference genomes of three relevant bat coronaviruses (bat-SL-CoVZC45 [accession no. MG772933.1], bat-SL-CoVZXC21, [accession no. MG772934.1], and RaTG13 [accession no. MN996532.1]) is also shown. Note that the pangolin metavirome similarity trace is not directly comparable to the bat coronavirus similarity traces, because the former uses read data for calculation, whereas the latter uses reference genomes.

**(v) Assessment of nucleotide similarity between SARS-CoV-2, pangolin metavirome reads, and closely related bat coronaviruses.** All pangolin metavirome reads that aligned to the SARS-CoV-2 genome with BWA-MEM after adapter trimming with cutadapt were used for calculation. The bat coronavirus genomes were aligned to the SARS-CoV-2 genome in a multiple sequence alignment using the web interface for Clustal Omega (https://www.ebi.ac.uk/Tools/msa/clustalo/) (9) with default settings. We note that sequence insertions with respect to the SARS-CoV-2 genome in either the pangolin metavirome reads or the bat coronavirus genomes are not accounted for in the similarity traces shown in Fig. 1b.

10.1128/mSphere.00160-20.1 FIG S1 (A) IGV snapshot of alignment across the spike protein and a zoom-in of the RBD, with the receptor binding motif (RBM) indicated. (B) IGV snapshot of a region within the RBD with apparently no coverage. The boxed regions of the reads denote soft-clipped portions. Download FIG S1, PDF file, 0.3 MB. Copyright © 2020 Wahba et al. 2020 Wahba et al. This content is distributed under the terms of the Creative Commons Attribution 4.0 International license.

**(vi) Regional assessment of synonymous and nonsynonymous mutations.** Although the incomplete nature of coverage in the pangolin metavirome data somewhat limits the application of measures such as normalized *dN*/*dS* (ratio of nonsynonymous to synonymous evolutionary changes) values, it remains possible to identify regions with the strongest matches of this inferred viral sequence with the human and bat homologs and to determine the distribution of synonymous and nonsynonymous variants in these regions. Details of this analysis are presented in Fig. S3 in the supplemental material.

**(vii) Accessibility of software.** Scripts used for the observations described in this communication are available at https://github.com/firelabsoftware/Metasearch2020.

### Findings.

To identify biological niches that might harbor viruses closely related to SARS-CoV-2, we searched through publicly available metavirome data sets. We were most interested in viruses with highly similar sequences, as these would likely be most useful in forming hypotheses about the origin and pathology of the recent human virus. We thus set a threshold requiring matching of a perfect 32-nucleotide segment with a granularity of 8 nucleotides in the search (i.e., interrogating the complete database of k-mers from the virus with k-mers starting at nucleotide 1, 9, 17, 25, 33 of each read from the metagenomic data for a perfect match). This would catch any perfect match of 39 nucleotides or greater (regardless of phasing relative to the 8-base search granularity), with some homologies as short as 32 nucleotides captured depending on the precise phasing of the read.

All metagenomic data sets with the keyword “virome” in NCBI SRA as of January 2020 were selected for analysis in a process that required approximately 2 days each for downloading and conversion to readable file formats and 1 day for searching by k-mer match on a desktop workstation computer (i7 8-core). Together the data sets included information from 9,014 NCBI Short Read Archive entries with (in total) 6.2 × 10^10^ individual reads and 8.4 × 1,012 bp. Despite the relatively large mass of data, the 32-nucleotide k-mer match remains a stringent measure, with spurious matches to the ∼30-kb SARS-CoV-2 genome expected at only 1 in 3 × 10^14^. Positive matches among the metagenomic data sets analyzed were relatively rare, with the vast majority of data sets (8,994/9,014 or 99.8%) showing no matched 32-mers to SARS-CoV-2. Of the data sets with matched k-mers, one was from a synthetic mixture of viral sequences that included a feline alphacoronavirus (10), while the remaining were all from vertebrate animal sources. The latter matches were from five studies: two bat-focused studies (11, 12), one bird-focused study (13), one study focused on small animals and rodents (14), and a study of pangolins (15) (Table 1).

**TABLE 1:**
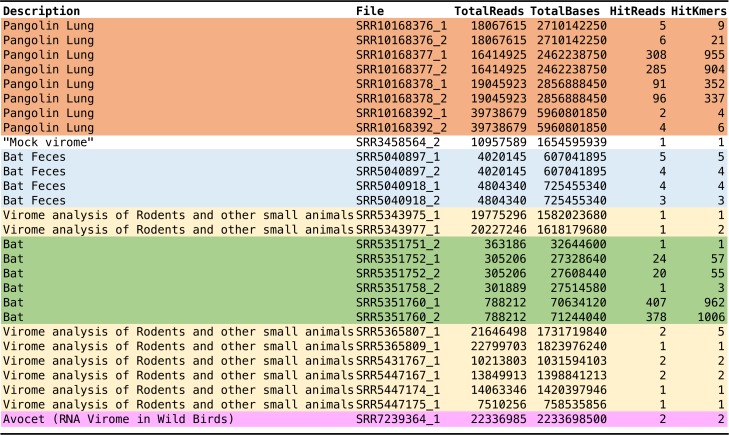
Metagenomic data sets with k = 32-mer matches to GenBank accession no. MN908947.3 (SARS-CoV-2)^*a*^

a Details of the search are described in the legend to Table S1 in the supplemental material.

10.1128/mSphere.00160-20.6 TABLE S1 Search results for SARS-CoV-2 and control sequences through all keyword virome NCBI SRA database entries as of 27 to 31 January 2020. Table S1 shows the results of a meta-metagenomic search of every data set from the SRA database as of January 2020 with the “virome” keyword. These data sets were probed with a set of viral query sequences as follows: MN908947.3 (SARS-CoV-2), cb1_chrUn:103428676-103444262, cb1_chrUn:16416583-16433048, and cb1_chrUn:16416583-16433048 (three sequences from the nematode C. briggsae), X51522.1 (bacteriophage P4), AF250364.2 (an arbitrary segment from an H1N1 influenza virus genome), AY603355.1 (a complete vaccinia virus genome; note that inspection of the vaccinia hits seems to indicate that some or all hits are from precise matches to sequence from an expression vector used to produce one or more enzyme used for cDNA library construction). Algorithms for search are as described in the “Search software” paragraph above, with source code provided at our GitHub site (https://github.com/firelabsoftware/Metasearch2020). The output format is as follows: column 1, Dataset_ReadNumber (e.g. SRRXXX_2 is the read 2 file from SRXXX); column 2, number of reads extracted and analyzed; column 3, number of bases analyzed; column 4, number of hits from the viral sampler query; column 5, total number of hit k-mers; column 6, summary of hits. Each element has a query name (e.g. X51522.1_phageP4), a position (e.g. p5012 starts at position 5012); a strand (“s” indicates a sense match starting at base, “a” indicates an antisense match, and a k-mer match count (e.g., m4 indicates that four different k-mers were matched every *n*th k-mer being checked [*n* is the search granularity]). Download Table S1, TXT file, 1.3 MB. Copyright © 2020 Wahba et al. 2020 Wahba et al. This content is distributed under the terms of the Creative Commons Attribution 4.0 International license.

The abundance and homology of viruses within a metagenomic sample are of considerable interest in interpreting possible characteristics of infection and relevance to the query virus. From the quick k-mer search, an initial indicator could be inferred from the number of matching reads and k-mer match counts for those reads (Table 1; see also Table S1 in the supplemental material). For the SARS-CoV-2 matches among the available metagenomic data sets, the strongest and most abundant matches in these analyses came from the pangolin lung metaviromes. The matches were observed throughout the SARS-CoV-2 query sequence, and many of the matching reads showed numerous matching 32-mer sequences. The vast majority of matches were in two lung samples—lung07 and lung 08—with small numbers of matches in two additional lung data sets, lung02 and lung09 (15). No matches were detected for seven additional lung data sets, and no matches were seen in eight spleen samples and a lymph node sample (15). Further analysis of coverage and homology through alignment of the metagenomic data sets revealed an extensive, if incomplete, coverage of the SARS-CoV-2 genome (Fig. 1a and Fig. S1A to C). Percent nucleotide similarity can be calculated for pangolin metavirome reads aligning to SARS-CoV-2 (Fig. 1b), and these segmental homologies consistently showed strong matches, approaching (but still overall weaker than) the similarity of the closest known bat coronavirus (RaTG13). A provisional comparison of synonymous differences at the nucleotide level between the pangolin reads, bat coronavirus RaTG13, and SARS-CoV-2 was also feasible for genes where pangolin sequences were available and readily aligned. Many synonymous (generally codon third base) changes were visible in such comparisons (Fig. S2 and S3). Comparisons of RaTG13 to SARS-CoV-2 revealed synonymous changes at 10% of conserved amino acid residues, while comparisons of the aggregate (but incomplete) pangolin reads indicated synonymous changes at 23% of conserved amino acid residues. Within the receptor binding domain (RBD) of the spike protein (16), these values are 26% and 34%, respectively.

10.1128/mSphere.00160-20.2 FIG S2 Nucleotide alignment of RBD between SARS-CoV-2, RaTG13, and pangolin. Observed comparisons from cDNA sequencing are shown for the indicated regions. Silent and nonsilent changes relative to SARS-CoV-2 are highlighted in green and red, respectively. Amino acid positions are indicated relative to the SARS-CoV-2 protein. Download FIG S2, PDF file, 0.08 MB. Copyright © 2020 Wahba et al. 2020 Wahba et al. This content is distributed under the terms of the Creative Commons Attribution 4.0 International license.

10.1128/mSphere.00160-20.3 FIG S3 Detailed plot of inferred substitutions. The plot shows incidence of inferred synonymous, nonsynonymous, and noncoding substitution from comparison of the RaTG13 assembly (P. Zhou, X. L. Yang, X. G. Wang, B. Hu, et al., Nature 579:270–273, 2020, https://doi.org/10.1038/s41586-020-2012-7) and a pangolin coronavirus scaffold, to the SARS-CoV-2 isolate from Wu et al. (F. Wu, S. Zhao, B. Yu, Y. M. Chen, et al., Nature 579:265–269, 2020, https://doi.org/10.1038/s41586-020-2008-3). The pangolin scaffold was generated from a BLAST alignment of SARS-CoV-2 mapping reads in SRR10168376/SRR10168377/SRR10168378. This plot addresses several challenges associated with the limited sequencing data available by attempting to provide the most favorable alignment of that sequence possible. To maximize sensitivity in detecting potential recombination, ambiguities in which two or more reads apparently disagreed (which were rare; approximately 1.9% of assigned bases) were resolved in favor of “no substitution” at any position if one read matched the SARS-CoV-2 genome. This provided a lower bound of variation, although regions covered by a single read are still subject to amplification and sequencing error. Near-perfect overlaps between reads from SRR10168376/SRR10168377/SRR10168378 argue that such error is relatively low, as agreement in those regions is 99.6%. The cumulative count of synonymous, nonsynonymous, and noncoding variants are per base pair and were taken over a 75-bp window (approximately 1/2 the read length) and then averaged over each 75-bp window with a weighting inversely proportional to distance. This resulted in the observed inverted spike for each individual variant. The top panel shows a plot for the full length of the virus. The bottom panel shows a plot for just the spike region (including the RBD). The total numbers of synonymous, nonsynonymous, and unchanged codons for the SARS-CoV-2 to pangolin comparison are 1,744, 640, and 5,841. respectively, with 1,530 SARS-CoV-2 amino acid residues absent in the limited pangolin alignments. For comparison, total numbers of synonymous, nonsynonymous, and unchanged codons for the SARS-CoV-2 to RaTG13 comparison are 936, 160, and 8,658. respectively, with one SARS-CoV-2 amino acid residue absent in the RaTG13 alignment. Download FIG S3, PDF file, 2.2 MB. Copyright © 2020 Wahba et al. 2020 Wahba et al. This content is distributed under the terms of the Creative Commons Attribution 4.0 International license.

The potential structural implications of protein sequence divergence in the RBD region of the spike protein were explored through combined sequence-directed structural alignment. The bat coronavirus RaTG13 RBD is markedly divergent relative to the SARS-CoV-2 RBD, with several amino acid differences located at the ACE-2 receptor-RBD interface (Fig. S4, with coordinate information shown in Text S1 in the supplemental material) (17). Thus, changes in these amino acid sequences, as previously described (17) and shown for comparison in Fig. S4 could be expected to influence interactions with the human ACE-2 receptor. In the case of the pangolin sequences, amino acid changes relative to the SARS-CoV-2 RBD seem to be, for the most part, located outside of the ACE-2 interface, with the exception of two residues (417 and 498) at the interface (17, 18). Overall, the SARS-CoV-2 and inferred pangolin virus amino acid sequences differ at seven positions in the RBD (residues 346, 372, 402, 417, 498, 519, and 529) (Fig. S4).

10.1128/mSphere.00160-20.4 FIG S4 Three-dimensional (3D) locations of differing amino acids in RaTG13 and inferred pangolin coronavirus RBDs relative to human SARS-CoV-2 RBD. To visualize possible structural positions of amino acid variations between SARS-CoV-2 RBD and homologous regions from bat coronavirus RaTG13 and pangolin reads, we first generated a provisional merged full 3D structure of SARS-CoV-2 spike and partial human ACE-2 receptor. A scaffold was created by performing a sequence-alignment directed structural superposition between PDB 2AJF (SARS-CoV RBD-ACE-2 complex), chain E (F. Li, W. Li, M. Farzan, and S. C. Harrison, Nature 309:1864–1868, 2005, https://doi.org/10.1126/science.1116480), and PDB 6VSB (SARS-CoV-2 spike trimer), chain A (D. Wrapp, N. Wang, K. S. Corbett, J. S. Goldsmith, et al., Science 367:1260–1263, 2020, https://doi.org/10.1126/science.abb2507). The hybrid coordinates were generated using UCSF Chimera and TK console in VMD. The provisional positions for this figure are available as supplemental files. (A) SARS-CoV-2 3D structure of the spike protein (transparent gray, blue, and cyan) with differing amino acids between SARS-CoV-2 and RaTG13 highlighted in red (residues 324, 346, 372, 403, 439, 440, 441, 443, 445, 449, 459, 478, 483, 484, 486, 490, 493, 494, 498, 501, 505, and 519). Notably, many residues are located at the interface between the RBD and the ACE-2 receptor (yellow) (J. Lan, J. Ge, J. Yu, S. Shan, et al., Nature, 2020, https://doi.org/10.1038/s41586-020-2180-5). Right-handed rotation is applied to the structure at the top of the panel to generate the view shown below. (B) Same structure in panel A with different amino acids between SARS-CoV-2 and the inferred pangolin coronavirus highlighted in red (residues 346, 372, 402, 417, 498, 519, and 529). Right-handed rotation is applied to the structure at the top of the panel to generate the view shown below. Download FIG S4, PDF file, 0.5 MB. Copyright © 2020 Wahba et al. 2020 Wahba et al. This content is distributed under the terms of the Creative Commons Attribution 4.0 International license.

10.1128/mSphere.00160-20.5 TEXT S1 Coordinate information used for Fig. S4. Supplemental text file 1 is a hybrid 3D coordinate file that was generated for the visualization in Fig. S4. The text-format document contains the original coordinates for PDB entry 6VSB (chains A, B, and C) with translocated and rotated coordinates for chain E and chain A in PBD entry 2AJF. The latter transformation, via sequence-directed structural alignment, results in PDB 2AJF chain E superimposed on untransformed coordinates for 6VSB chain A. Download Text S1, TXT file, 2.3 MB. Copyright © 2020 Wahba et al. 2020 Wahba et al. This content is distributed under the terms of the Creative Commons Attribution 4.0 International license.

### Conclusions.

Meta-metagenomic searching can provide unique opportunities to understand the distribution of nucleic acid sequences in diverse environmental niches. As metagenomic data sets proliferate and as both the need and capability to identify pathogenic agents through sequencing increase, meta-metagenomic searching may prove extremely useful in tracing the origins and spreading of causative agents. In the example we present in this paper, such a search identifies a number of niches with sequences matching the genome of the SARS-CoV-2 virus. These analyses raise a number of relevant points for the origin of SARS-CoV-2. Before describing the details of these points, however, it is important to stress that while environmental, clinical, and animal-based sequencing is valuable in understanding how viruses traverse the animal ecosphere, static sequence distributions cannot be used to construct the full transmission history of a virus among different biological niches. So even if the closest relative of a virus-causing disease in species X were to be found in species Y, we cannot define the source of the outbreak or the direction(s) of transmission. As some viruses may move more than once between hosts, the sequence of a genome at any time may reflect a history of selection and drift in several different host species. This point is also accentuated in the microcosm of our searches for this work. When we originally obtained the SARS-CoV-2 sequence from the posted work of Wu et al. (3), we recapitulated their result that bat-SL-CoVZC45 was the closest related sequence in NCBI’s nonredundant (nr/nt) database. In our screen of metavirome data sets, we observed several pangolin metavirome sequences, which were not in the NCBI nr/nt database at the time, that are more closely related to SARS-CoV-2 than bat-SL-CoVZC45. An assumption that the closest relative of a sequence identifies the origin would at that point have transferred the extant model to zoonosis from pangolin instead of bat. To complicate such a model, an additional study from Zhou et al. (4) described a previously unpublished coronavirus sequence, designated RaTG13 with much stronger homology to SARS-CoV-2 than either bat-SL-CoVZC45 or the pangolin reads from Liu et al. (15). While this observation certainly shifts the discussion (legitimately) toward a possible bat-borne intermediate in the chain leading to SARS-CoV-2, it remains difficult to determine if any of these are true intermediates in the chain of infectivity.

The match of SARS-CoV-2 to the pangolin coronavirus sequences also enables a link to substantial context on the pangolin samples from Liu et al. (15), with information on the source of the rescued animals (from smuggling activity), the nature of their deaths despite rescue efforts, the potential presence of other viruses in the same whole-lung tissue, and the accompanying gross pathology. The pangolins appear to have died from lung-related illness, which may have involved a SARS-CoV-2-homologous virus. Notably, however, two of the deceased pangolin lungs had much lower SARS-CoV-2 signals, while seven showed no signal, with sequencing depths in the various lungs roughly comparable. Although it remains possible that the SARS-CoV-2-like coronavirus was the primary cause of death for these animals, it is also possible (as noted by Liu et al. [15]) that the virus was simply present in the tissue, with mortality due to another virus, a combination of infectious agents, or other exposures.

During the course of this work, the homology between SARS-CoV-2 and pangolin coronavirus sequences in a particular genomic subregion was also noted and discussed in an online forum (“Virological.org”) with some extremely valuable analyses and insights. Matthew Wong and colleagues bring up the homology to the pangolin metagenomic data sets in this thread and appear to have encountered it through a more targeted search than ours (this study has since been posted online on bioRxiv [19]). As noted by Wong et al. (19), the spike region includes a segment of ∼200 nucleotides encompassing the RBD where the inferred divergence between RaTG13 and SARS-CoV-2 dramatically increases. This region is of interest, as it is a key determinant of viral host range and under heavy selection (20). The observed spike region divergence indeed includes a substantial set of nonsynonymous differences (Fig. S2 and S3). Notably, while reads from the pangolin lung data sets mapped to this region do not show a similar increase in variation relative to SARS-CoV-2, we also did not observe a significant drop in variation between SARS-CoV-2 and pangolin sequences in this region (Fig. S2 and S3). Instead, variation in the region is comparable to numerous other conserved regions of the spike and to the viral genome as a whole. While Wong et al. (19) and others (21–28) raised the model that recombination occurred in the RBD region in the derivation of SARS-CoV-2, the lack of a singular dip in the landscape of pangolin-SARS-CoV-2 variation in the region would seem counterintuitive were SARS-CoV-2 a result of a localized recombination between a close relative of RaTG13 and a close relative of the putative pangolin coronaviruses under consideration. Thus alternative models for the observed sequence variation seem important to consider and indeed parsimonious, including that of selection acting on the RaTG13 sequences in bats or another intermediate host resulting in a rapid variation of the amino acids at the highly critical virus-receptor interface. Overall, definitive conclusions regarding the origins of SARS-CoV-2 or other coronaviruses will remain difficult with limited sequencing data and without knowledge of evolutionary trajectories in different lineages (29, 30).

A number of literature contributions now discuss the potential role for bats, pangolins, and other possible progenitor/intermediate species in derivation of SARS-CoV-2 from different approaches and perspectives, with a diversity of approaches and interpretations in understanding the origin of the virus. In particular, there has been extensive discussion and debate about the possible pangolin origin of SARS-CoV-2 (19, 21–28, 31–41). These studies provide useful insights into the evolution of SARS-CoV-2 but have limitations and uncertainty in drawing conclusions regarding the viral origin, as most studies were mainly performed through sequence-based comparison and simulation. Thus, better understanding of the current pandemic requires additional information on investigational, experimental, and epidemiological levels that may resolve questions of origin and of preventing the reemergence of SARS-CoV-2 and other pathogens. Nevertheless, the availability of numerous paths (both targeted and agnostic) toward identification of natural niches for pathogenic sequences, including our meta-metagenomic search, will remain useful to the scientific community and to public health, as will vigorous sharing of ideas, data, and discussion of potential origins and modes of spread for epidemic pathogens.
