# Supplementary figures and images for: An Extensive Meta-Metagenomic Search Identifies SARS-CoV-2-Homologous Sequences in Pangolin Lung Viromes
Source: mSphere. 2020 May 6;5(3):e00160-20. doi: 10.1128/mSphere.00160-20 (PMC7203451; doi:10.1128/mSphere.00160-20)

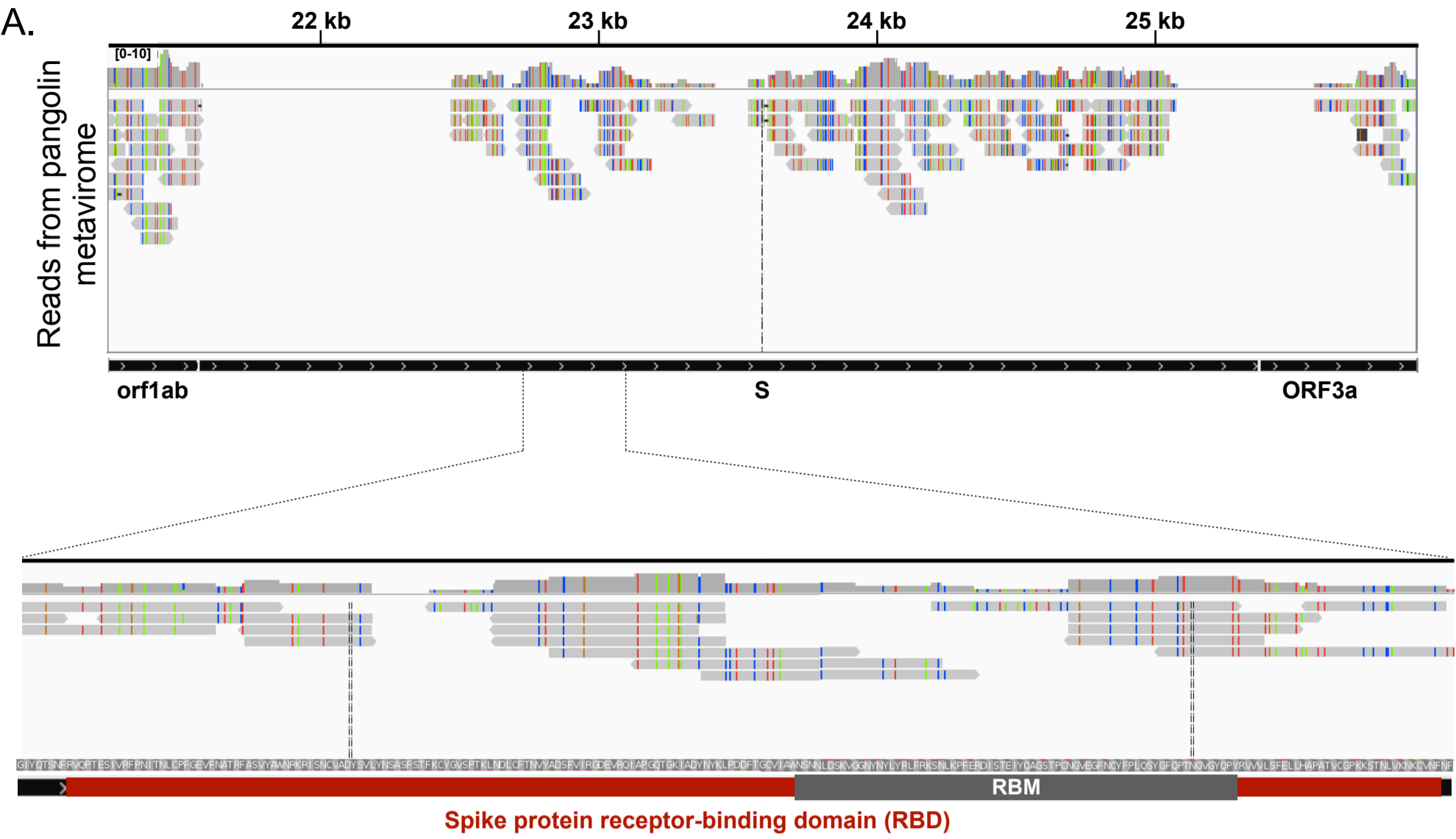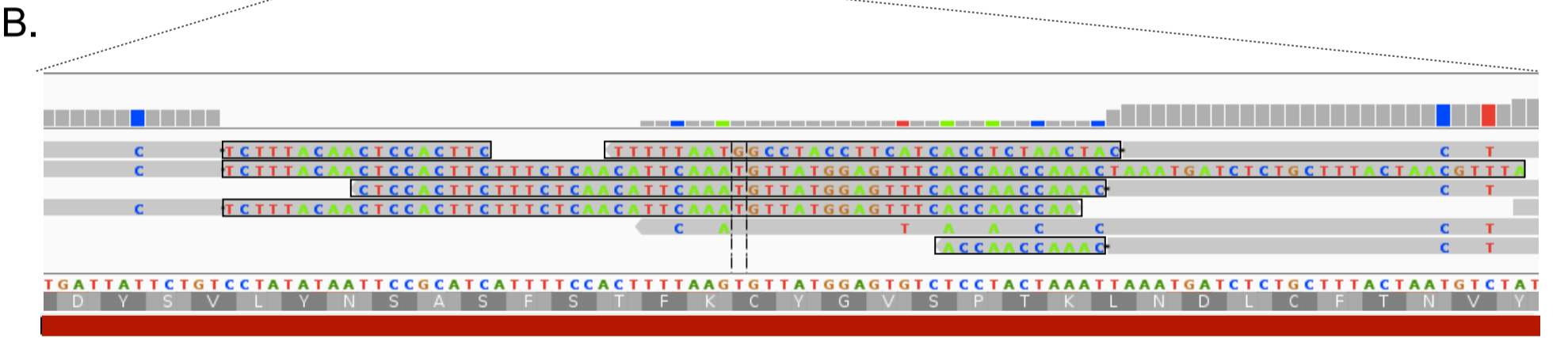

Supplement: FIG S1 [file mSphere.00160-20-sf001.pdf]

**A.**

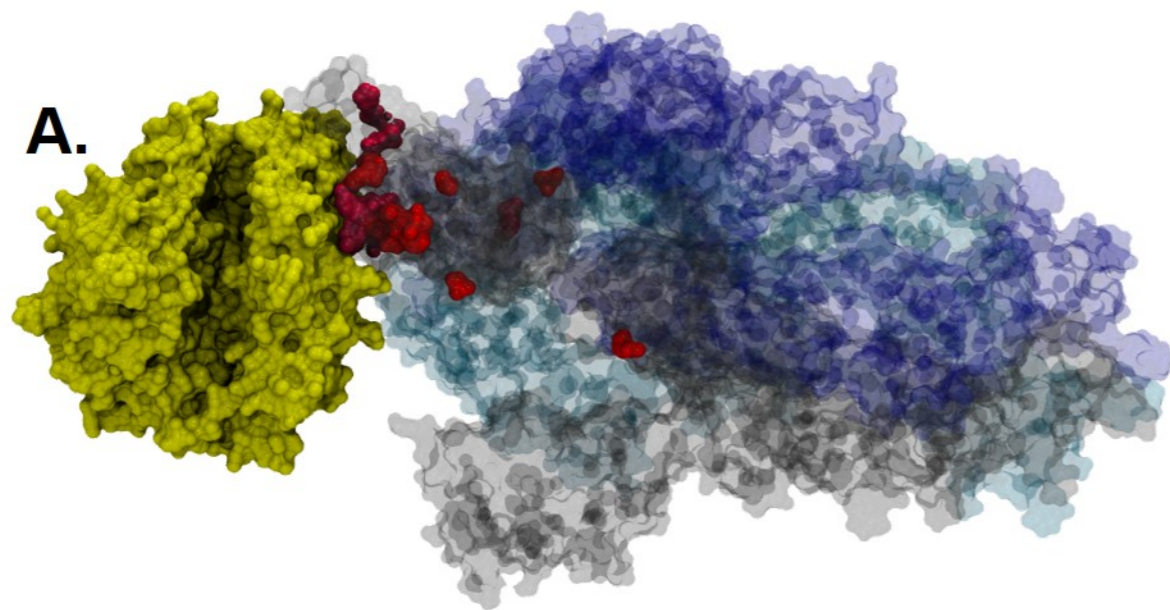

**B.**

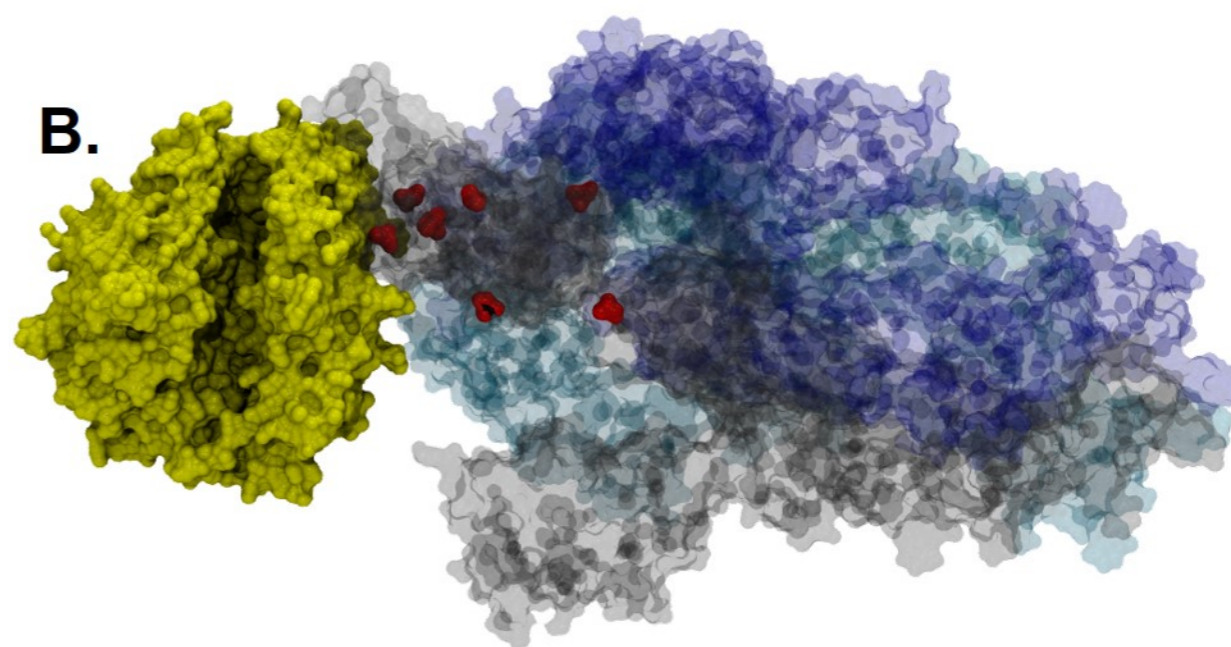

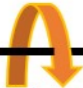  
Right-handed 60°

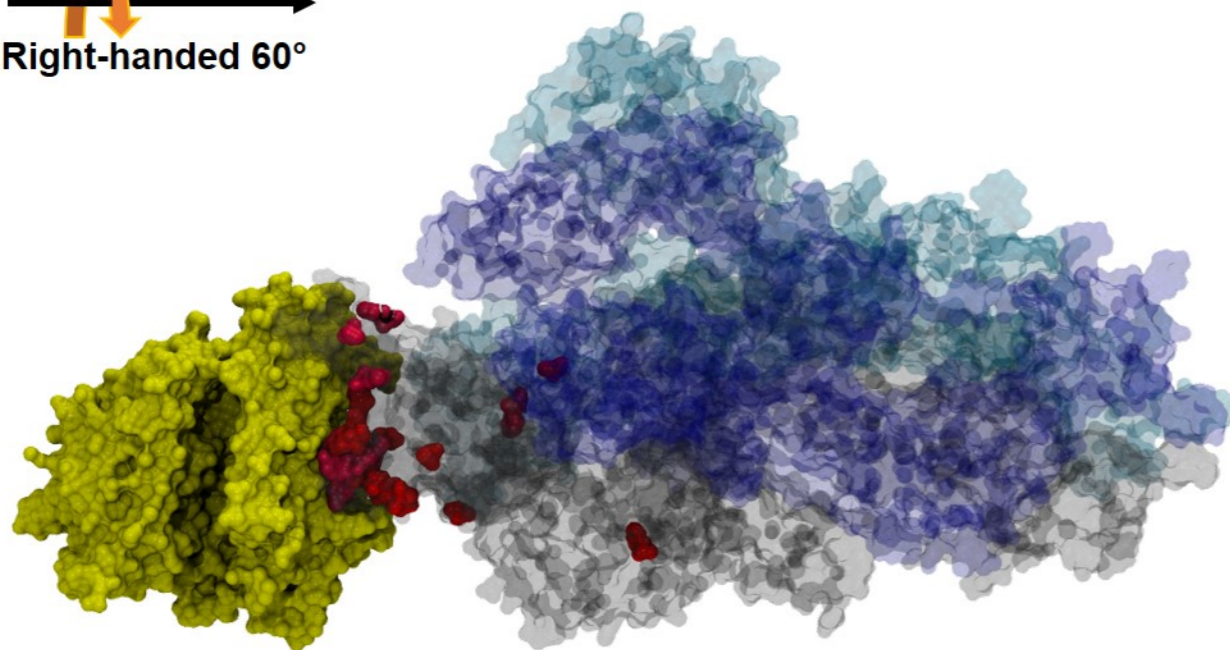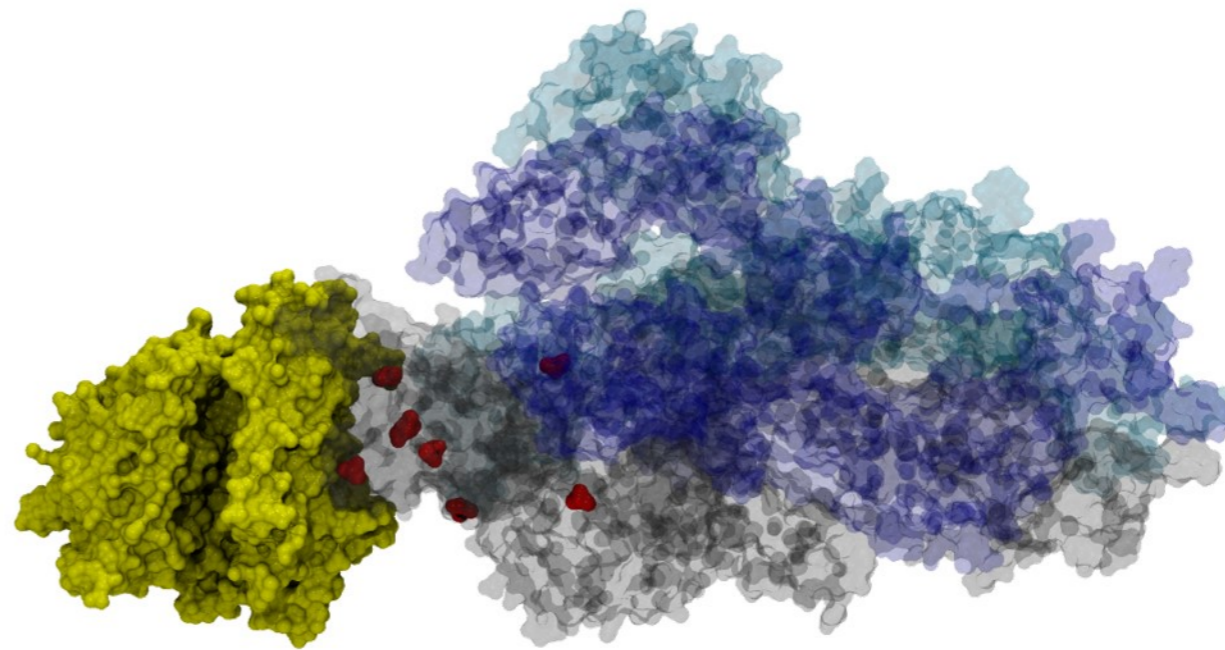

Supplement: FIG S4 [file mSphere.00160-20-sf004.pdf]
