# Supplementary material for: An Extensive Meta-Metagenomic Search Identifies SARS-CoV-2-Homologous Sequences in Pangolin Lung Viromes
Source: mSphere. 2020 May 6;5(3):e00160-20. doi: 10.1128/mSphere.00160-20 (PMC7203451; doi:10.1128/mSphere.00160-20)
Supplement: FIG S2 [file mSphere.00160-20-sf002.pdf]

```
Human     AGAGTCCAACCAACAGAATCTATTGTTAGATTTCCTAATATTACAAACTTGTGCCCTTTTGGTGAAGTTTTTAACGCCACCACATTCGCATCAGTTTATGCTTGGAACAGAAAGAGAATT
RaTG13    AGAGTCCAACCAACAGATTCTATTGTTAGATTCCCAAATATTACAAACTTATGTCCTTTTGGTGAAGTTTTTAACGCCACCAGATTTGCATCTGTTTATGCTTGGAACAGGAAGAGAATC
Pangolin  AGAGTTCAACCAACTGAATCTATAGTTAGGTTTCCAAATATTACAAACTTATGCCCTTTTGGTGAAGTTTTCAATGCAACCACTTTTGCATCTGTTTATGCTTGGAATAGAAAGAGAATC
          ***** ******** ** ***** ***** ** ** ************** ** ***************** ** ** ****  ** ***** ************** ** ********
Amino acid
coordinates  319                                                                                                          358

Human     AGCAACTGTGTTGCTGATTATTCTGTCCTATATAATTCCGCATCATTTTCCACTTTTAAGTGTTATGGAGTGTCTCCTACTAAATTAAATGATCTCTGCTTTACTAATGTCTATGCAGAT
RaTG13    AGCAACTGTGTTGCTGATTACTCTGTCCTATATAATTCCACTTCATTTTCTACCTTTAAATGTTATGGAGTGTCTCCTACTAAATTAAATGATCTCTGCTTTACTAATGTTTATGCAGAC
Pangolin  AGTAACTGTGTTGCTGATTACTCTGTTCTTTACAACTCCACTTCTTTCTCAACATTCAAATGTTATGGAGTTTCACCAACCAAACTAAATGATCTCTGCTTTACTAACGTTTATGCAGAC
          ** **************** ***** ** ** ** *** * ** ** ** ** ** ** *********** ** ** ** *** ********************* ** ********
Amino acid
coordinates  359                                                                                                          398

Human     TCATTTGTAATTAGAGGTGATGAAGTCAGACAAATCGCTCCAGGGCAAACTGGAAAGATTGCTGATTATAATTATAAATTACCAGATGATTTTACAGGCTGCGTTATAGCTTGGAATTCT
RaTG13    TCATTTGTGATTACAGGTGATGAAGTCAGACAAATTGCGCCAGGACAAACTGGAAAGATTGCTGACTACAATTATAAACTACCAGATGATTTTACTGGTTGTGTTATAGCTTGGAATTCT
Pangolin  TCATTTGTAGTTAGAGGTGATGAAGTCAGACAAATTGCTCCAGGACAAACAGGAAGAATTGCTGACTATAATTATAAACTCCCTGATGATTTCACAGGTTGTGTAATAGCTTGGAATTCT
          ********  *** ******************** ** ***** ***** ****  ******** ** ********* * ** ******** ** ** ** ** ***************
Amino acid
coordinates  399                                                                                                          438

Human     AACAATCTTGATTCTAAGGTTGGTGGTAATTATAATTACCTGTATAGATTGTTTAGGAAGTCTAATCTCAAACCTTTTGAGAGAGATATTTCAACTGAAATCTATCAGGCCGGTAGCACA
RaTG13    AAGCATATTGATGCAAAAGAGGCGGTAATTTTAACTATCTTTACCGTCTCTTTAGAAAAGCTAATCTTAAACCCTTTGAGAGGGATATCTCAACTGAAATTTACCAAGCAGGCAGCAAA
Pangolin  AACAACCTTGATTCTAAGGTTGGTGGTAATTATAACTACCTTTATAGATTGTTTAGAAAGTCCAACCTCAAACCTTTTGAACGAGACATTTCTACAGAAATATACCAAGCTGGTAGTACA
          **   *   ***** * ** *   ** ******* *** ** ** **   *   * ***** **   * ** ** ***** *****   * ** ** ** ** ***** ** ** ** ** ** * *
Amino acid
coordinates  439                                                                                                          478

Human     CCTTGTAATGGTGTTGAAGGTTTTAATTGTTACTTTCCTTTACAATCATATGGTTTCCAACCCACTAATGGTGTTGGTTACCAACCATACAGAGTAGTAGTACTTTCTTTTGAACTTCTA
RaTG13    CCTTGTAATGGTCAAACTGGTCTAAATTGCTACTACCCACTTTATAGATATGGATTTTACCCTACTGATGGTGTTGGTCACCAACCTTATAGGGTAGTAGTACTTTCTTTTGAACTTCTA
Pangolin  CCCTGCAATGGGGTTGAAGGTTTTAACTGTTACTTTCCTCTACAATCTTATGGTTTCCACCCTACTAATGGTGTTGGTTACCAACCTTATAGAGTAGTAGTATTGTCATTTGAACTTTTA
          ** ** *****        *** * ** ** ****  **   *   *    ***** **   * ** *** *********** ******* ** ** ********* * ** ********* **
Amino acid
coordinates  479                                                                                                          518

Human     CATGCACCAGCAACTGTTTGTGGACCTAAAAAGTCTACTAATTTGGTTAAAAACAAATGTGTCAATTTC
RaTG13    AATGCACCAGCAACTGTTTGTGGACCTAAGAAGTCTACTAACTTGGTTAAAAATAAATGTGTCAATTTC
Pangolin  AATGCACCTGCTACTGTTTGTGGACCTAAACAGTCCACTAACCTAGTTAAAAACAAATGTGTCAACTTC
           ******* ** ****************  **** *****  * ******** *********** ***
Amino acid
coordinates  519                                                             541
```
