# Supplementary material for: An Extensive Meta-Metagenomic Search Identifies SARS-CoV-2-Homologous Sequences in Pangolin Lung Viromes
Source: mSphere. 2020 May 6;5(3):e00160-20. doi: 10.1128/mSphere.00160-20 (PMC7203451; doi:10.1128/mSphere.00160-20)
Supplement: FIG S3 [file mSphere.00160-20-sf003.pdf]

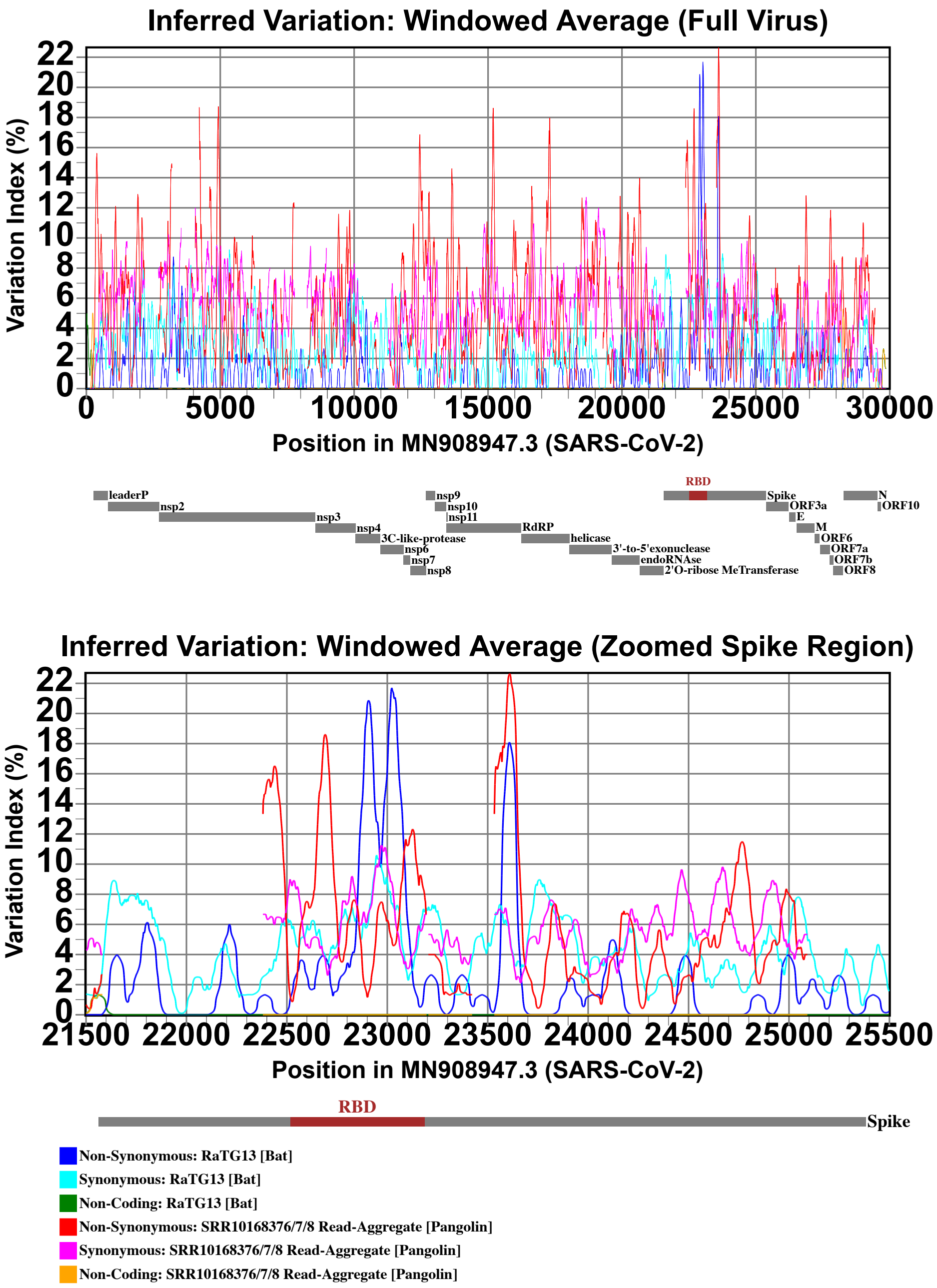

Inferred Variation: Windowed Average (Full Virus)
Variation Index (%)
Position in MN908947.3 (SARS-CoV-2)
0 5000 10000 15000 20000 25000 30000
0 2 4 6 8 10 12 14 16 18 20 22
RBD
leaderP
nsp2
nsp3
nsp4
3C-like-protease
nsp6
nsp7
nsp8
nsp9
nsp10
nsp11
RdRP
helicase
3'-to-5'exonuclease
endoRNAse
2'O-ribose MeTransferase
Spike
ORF3a
E
M
ORF6
ORF7a
ORF7b
ORF8
N
ORF10
Inferred Variation: Windowed Average (Zoomed Spike Region)
Variation Index (%)
Position in MN908947.3 (SARS-CoV-2)
21500 22000 22500 23000 23500 24000 24500 25000 25500
0 2 4 6 8 10 12 14 16 18 20 22
RBD
Spike
Non-Synonymous: RaTG13 [Bat]
Synonymous: RaTG13 [Bat]
Non-Coding: RaTG13 [Bat]
Non-Synonymous: SRR10168376/7/8 Read-Aggregate [Pangolin]
Synonymous: SRR10168376/7/8 Read-Aggregate [Pangolin]
Non-Coding: SRR10168376/7/8 Read-Aggregate [Pangolin]
